# B Cells at the Cross-Roads of Autoimmune Diseases and Auto-Inflammatory Syndromes

**DOI:** 10.3390/cells11244025

**Published:** 2022-12-12

**Authors:** Moncef Zouali

**Affiliations:** Graduate Institute of Biomedical Sciences, China Medical University, Taichung City 404, Taiwan; moncef.zouali@wanadoo.fr

**Keywords:** B cell, autoinflammation, autoimmunity, mevalonate kinase deficiency syndrome, Kawasaki syndrome, inflammatory bone disorders, Schnitzler syndrome, Neuro-Behçet’s disease, neuromyelitis optica spectrum

## Abstract

Whereas autoimmune diseases are mediated primarily by T and B cells, auto-inflammatory syndromes (AIFS) involve natural killer cells, macrophages, mast cells, dendritic cells, different granulocyte subsets and complement components. In contrast to autoimmune diseases, the immune response of patients with AIFS is not associated with a breakdown of immune tolerance to self-antigens. Focusing on B lymphocyte subsets, this article offers a fresh perspective on the multiple cross-talks between both branches of innate and adaptive immunity in mounting coordinated signals that lead to AIFS. By virtue of their potential to play a role in adaptive immunity and to exert innate-like functions, B cells can be involved in both promoting inflammation and mitigating auto-inflammation in disorders that include mevalonate kinase deficiency syndrome, Kawasaki syndrome, inflammatory bone disorders, Schnitzler syndrome, Neuro-Behçet’s disease, and neuromyelitis optica spectrum disorder. Since there is a significant overlap between the pathogenic trajectories that culminate in autoimmune diseases, or AIFS, a more detailed understanding of their respective roles in the development of inflammation could lead to designing novel therapeutic avenues.

## 1. Introduction

In response to exposure to invading pathogens or to endogenous stimuli derived from damaged cells, the immune system mounts inflammatory processes that can lead to tissue repair or pathology. Deciphering the mechanisms and roles of inflammation during physiological and pathological immune responses recently has undergone important advances, driven, in part, by single-cell analysis and high-throughput profiling. In this research arena, studies of a particular group of diseases, called auto-inflammatory syndromes (AIFS), are providing important insights into the links between inflammatory processes and human pathology.

Two decades ago, studies of a subset of human diseases characterized by systemic inflammation and involving cells of the innate immune system, but lacking attributes of autoimmune diseases, led to the concept of autoinflammation. Specifically, investigation of autosomal dominant periodic fever syndromes allowed identification of mis-sense mutations of the gene encoding tumor necrosis factor receptor (TNFR1) associated with low levels of soluble plasma TNFR1 [1]. It was proposed that deregulation of innate immunity pathways can give rise to an auto-inflammatory phenotype. This view implied a clear distinction between autoimmune disease and AIFS, the latter being characterized by deregulation of the innate immune system that causes a hyper-inflammatory state. Neutrophils and monocytes are thought to be the central effector cells, and the adaptive branch of immunity, including B cells and autoantibody production, is considered to play a marginal role, if any [2,3]. On the other hand, loss of immune tolerance to self-antigens is a hallmark of autoimmune diseases, and activation of T cells and B cells can give rise to the production of autoantibodies and autoreactive T cells, resulting in tissue damage of multiple organs [4].

AIFS can be categorized in two subgroups. First, monogenic autoinflammatory disorders result from inborn monogenetic mutations that affect the innate immune system and culminate in undesirable inflammation. They include autosomal dominant TNF receptor-associated periodic syndrome (TRAPS) [5], Familial Mediterranean Fever (FMF), Pyrin-Associated Autoinflammation with Neutrophilic Dermatosis (PAAND), Cryopyrin-associated periodic syndromes (CAPS), NLRP12-associated disorder (NLRP12AD), and mevalonate kinase deficiency (MKD). These conditions are characterized by recurrent febrile episodes, but no infectious agent can be identified [2,3]. Second, polygenic, complex AIFS comprise disorders of unknown etiology, including hereditary and multifactorial disorders. Clinical manifestations are nonspecific, and include arthritis, cutaneous lesions, fever, abdominal pain, arthritis, cutaneous lesions, central nervous system (CNS) involvement and hearing loss. Complex AIFS comprise idiopathic recurrent pericarditis (IRP), adult-onset Still disease (AOSD), Schnitzler syndrome, crystal-induced arthropathies, systemic juvenile idiopathic arthritis (sJIA), Kawasaki disease, idiopathic recurrent pericarditis (IRP), Behçet’ disease and gout, a disorder in which the central event of inflammation is the activation of white blood cells by monosodium urate crystals [6].

Whereas autoimmune diseases are mediated primarily by T and B cells, AIFS involve natural killer cells, macrophages, mast cells, dendritic cells, different granulocyte subsets and complement components. In contrast to autoimmune disease, the immune response of patients with AIFS does not target specific antigens, and the systemic chronic inflammation is not associated with a breakdown of immune tolerance to self-antigens. Additionally, environmental factors, such as mechanical stress, temperature change, or infection, can be responsible for the development of an inflammatory cascade in susceptible genetic backgrounds [7]. However, as is discussed below, recent observations suggest that a continuum of innate and adaptive mechanisms underlies the pathogenesis of complex AIFS [8].

## 2. Altered Functions of B Lymphocytes in Autoinflammatory Disorders

Post-translational modification by ubiquitination/deubiquitination is involved in regulation of several processes, including DNA-repair, protein degradation, and endocytosis [9]. In particular, ubiquitination is implicated in regulation of the canonical NF-kB signaling pathway and influences NLRP3 inflammasome activation [10]. It also stabilizes molecular complexes that promote cytokine signaling, including the IL-1 receptor (IL-1R), with the linear ubiquitin chain assembly complex (LUBAC) maintaining the stability of several cytokine receptors. This complex is formed by heme-oxidized IRP2 ubiquitin ligase 1L (HOIL-1), together with other proteins. Recent studies showed that certain AIFS result from alterations of the ubiquitin-proteasome [11]. For example, genetic loss of ubiquitin-proteasome function leads to proteasome-associated autoinflammatory syndromes (PRAASs) in humans [12]. In addition, several alterations of the LUBAC complex have been found to lead to development of AIFS, termed ubiquitinopathies [13]. Patients deficient in HOIL show hepatosplenomegaly and lymphadenopathy. Remarkably, they also manifest severe alterations of B cell functions and compromised NF-kB responses in B cells, causing recurrent bacterial infections [13,14].

Rubinstein-Taybi syndrome (RSTS) is a rare genetic disorder characterized by a typical facial dysmorphism and several additional phenotypical signs. In studies of an international cohort of 97 patients with RSTS, autoinflammatory complications and lymphoproliferation were observed in the majority of cases. In addition, hypogammaglobulinemia was associated with low B cell counts and reduction of switched memory B cell numbers [15]. These dysfunctions, affecting mostly B cells, warrant further detailed immunological assessment in these patients.

## 3. Multiple Roles of B Cells in Autoinflammatory Disorders

Restriction of inflammatory responses depends, at least in part, on cholesterol metabolism [16]. As a result, patients exhibiting mutations in genes implicated in cholesterol metabolism pathways develop severe and recurring auto-inflammation, together with abnormal B cell responses [17]. By modulating TLR9 signaling, cholesterol metabolism is essential for the induction of human IL-10-secreting regulatory B cells known to play a role in different contexts, including cancer, infection, and autoimmune disease [18]. Downstream of TLR9 engagement, BLIMP1 acts as a transcriptional regulator of IL-10 expression. In humans, loss-of-function mutations in the gene encoding mevalonate kinase underly development of mevalonate kinase deficiency (MKD) syndrome, also called Hyper IgD syndrome. MKD patients are typically diagnosed in early childhood with high serum circulating levels of mevalonic acid, IgA, IgD, and IL-1β [17]. Consequently, the ability of MKD patients to convert mevalonate to mevalonate-5-phosphate is severely impaired, and the diseased subjects exhibit poor regulatory B cell responses [19], significant B cell cytopenia, hypogammaglobulinemia, and an autoinflammatory symptomatology [20]. A combination of prednisone, azathioprine, and intravenous immunoglobulins reduces the incidence and severity of the febrile attacks. In addition to a reduced ability to produce IL-10, a functional impairment in restricting T cell responses has been observed in these patients [19]. It is possible that this B cell defect contributes to the relapsing and remitting episodes of the disease.

Kawasaki syndrome (KS) is responsible for an acquired cardiac disorder in children [21]. It is characterized by fever and coronary artery inflammation and aneurysm. The diagnosis is based on four out of five clinical criteria, and several pathogenic mechanisms have been proposed, including post-infectious autoimmune inflammation [22]. Several animal models of coronary arteritis have been used to gain insight into the pathogenesis of KD [23]. In one of them, a single intraperitoneal injection of a cell wall extract from *Lactobacillus casei* triggered development of proximal coronary arteritis that exhibits striking similarities in the histopathology and kinetics with human KS. In this model, both innate and adaptive immunity were found to be essential for development of coronary lesions [24].

The implications of B cells in KS pathogenesis comes from human genetic and histopathogy studies. First, genome-wide association studies suggest that genes involved in B cell activation are implicated [25,26]. In both Japanese and Taiwanese KS patients, significant associations in the FAM167A-BLK region at 8p22–23 and in the CD40 region at 20q13 were identified [25,26]. BLK codes for the Src family kinase Blk, which is responsible for downstream signaling of the B cell receptor and affects the proliferation/differentiation and tolerance of B cells [27,28]. Blk is also required for T-cell-mediated proinflammatory cytokine production [29]. The cell surface receptor CD40 is expressed on antigen-presenting cells, such as B cells, and plays a key role in proliferation, differentiation, and activation of B cells, and in T cell-dependent immune responses [30]. Thus, the kinase Blk and the receptor CD40, which play key roles in the B cell compartment, seem to be prominent in determining the risk for KS. 

Second, progress has been made in understanding the pathogenesis of KS through examination of tissue samples from fatal cases. Studies of human autopsy specimens revealed early infiltration by neutrophils, followed by mixed lymphocytes, plasma cells and macrophages [31,32]. The B cell and plasma cell infiltration in specimens from KS patients suggest that B lymphocytes could play a role in tissue injury. They could be the result of the activity of a B cell superantigen [13,33] or of an infectious agent. They also could represent the product of B cell hyperactivation by ligands that can lead to polyclonal activation.

Initially, several groups suggested that KS is caused by multiple infectious agents, and several microorganisms have been isolated from patients with KS. Consistently, sequence analysis of the IgM transcripts expressed by peripheral B cells of KS patients in the acute phase of the disease revealed oligoclonal expansions of Ig variable region genes, suggesting that KS is caused by stimulations in response to an antigenic stimulus [34]. In further investigations, oligoclonal IgA plasma cells were documented to infiltrate inflamed tissues, including the coronary arteries of patients with acute KD [35]. Remarkably, this infiltration contrasts with the decrease of absolute numbers of IgA B lymphocytes in peripheral blood of patients with acute KS [36]. Further characterization of the Ig heavy-chain-genes present in the arterial wall of children who had died of acute KS exhibited a restricted pattern of CDR3 usage and somatic mutation, a marker of an antigen-driven response [37]. Synthetic antibody versions of these oligoclonal KS antibodies are bound to an antigen present in inflamed acute KS ciliated bronchial epithelium [38].

These observations are consistent with the view that, following infection of ciliated bronchial epithelium of the respiratory tract by a pathogen, intra-cytoplasmic inclusion bodies are formed. In order to neutralize the presence of the pathogen, antigen-specific plasma cells infiltrate the infected tissue. However, a collateral effect of this infiltration may lead to damage of the coronary arteries by products of activated lymphocytes and, possibly, other inflammatory cells. A better identification of the B cell subsets present at the site of inflammation could provide further insight into the physiopathology of KS.

Relevant to innate immunity and auto-inflammation are the inflammasomes, multiprotein cytoplasmic complexes that act as regulators of immune recognition trajectories, including viral, bacterial, and fungal pathogens [39]. Inflammasome activation leads to IL-1β and IL-18 maturation, a key process in the host defense against potential threats. Not surprisingly, deregulation of NLRP3 has been linked to the physio-pathology of AIFS, as well as several degenerative and metabolic disorders. For example, mutations in genes coding for components of the NLRP3 inflammasome lead to an AIFS called Cryopyrin-Associated Periodic Syndromes (CAPS) [40]. In human B cells, NLRP3 has been reported to be essential for pro-inflammatory cytokine secretion, and IL-1β secretion was modulated by NLRP3 and involved potassium efflux and Caspase-1 [41]. Consistently, ablation of the NLRP3 inflammasome in mice leads to altered B cell development in the bone marrow, and distorted expression of B cell subsets that play innate-like functions, i.e., marginal zone B cells in the spleen and B-1a cells in the peritoneal cavity, indicating that the inflammasome plays a role in B cell development, homing, and retention in lymphoid organs [42]. Since the NLRP3 inflammasome acts as a modulator of B lymphocyte functions, further work is required to understand this role in auto-inflammatory disorders.

Patients affected with Schnitzler syndrome manifest cardinal features of AIFS, but also have a monoclonal IgM gammopathy associated with a kappa light-chain in over 98% of cases [43], suggesting that B cells could play a role. To determine whether B cells exhibit shared clonality, deep sequencing of the immunoglobulin variable region genes from Schnitzler syndrome patients has been performed, allowing characterization of the immunoglobulin variable region gene usage, and the length and amino acid composition of the third hypervriable regions [44]. The investigators found evidence of various degrees of B cell clonality in each individual case. However, they could not document shared B cell clonality among all the patients studied. This study suggests that the adaptive branch of the immune system plays a role in this autoinflammatory condition. However, since the investigators undertook sequencing of immunoglobulin genes of peripheral blood B cells, it is likely that resident B cell clones present in the bone marrow or secondary lymphoid organs have been missed. Further longitudinal studies of resident B cell clones in these patients could provide insight into the role of B cells in Schnitzler syndrome.

**Figure 1:**
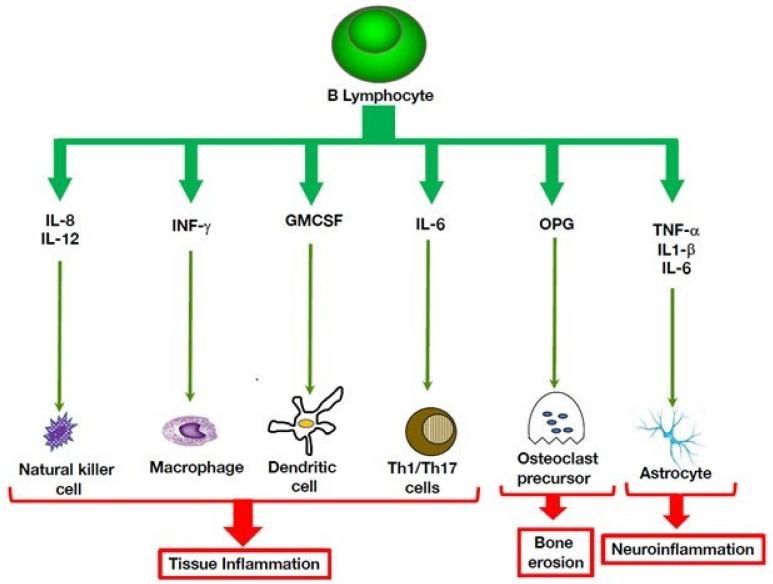
B cell cytokine-mediated pro-inflammatory pathways that act on innate immunity. B cells can exert multiple immune activities that promote inflammatory processes by producing cytokines that act on immune cells that mediate inflammation, such as macrophages and natural killer cells. Through their interactions with the skeletal system, they can promote bone erosion 46. In the central nervous system, their potential to secrete inflammatory cytokines can aggravate neuroinflammation.

Inflammatory bone disorders are characterized by production of pro-inflammatory cytokines and a variety of danger-associated molecular patterns (DAMPs), such as purine metabolites and fatty acids [45]. It is possible that autoinflammation plays a role in systemic bone loss associated with various rheumatic and musculoskeletal diseases, potentially leading to osteoporosis and fragility fractures. In support of this view, B lymphocytes have the potential to engage in multi-directional cross-talk with cells of the skeletal system, including osteocytes, osteoclasts and osteoblasts [46]. For example, osteoprotegerin produced by B cells is key in regulating bone remodeling mediated by the RANK-RANK-L pair (Figure 1). Given the tight relationships between B lymphocytes and the skeletal system, the role of B cells in autoinflammation in bone disorders deserves further consideration.

Behçet’s disease (BD) is an auto-inflammatory vasculitis that affects predominantly the mucosa, skin and eyes [47]. In addition to elevated serum cytokine levels, such as IL-1β, TNF-α, and IL-18, there is a distorted expression of toll-like receptors (TLR). Cells that express these receptors can recognize conserved motifs present on pathogens, termed pathogen-associated molecular patterns (PAMPs) and DAMPs, which lead to activation of a signaling cascade that culminates in production of pro-inflammatory cytokines, including TNF-α [45]. A recent study of TLR expression in Behçet’s disease revealed deregulated expression of different cell subsets, including monocytes, granulocytes and T cells [8]. In addition, TLR1 and TLR2, TLR4 and TLR5 were abnormally expressed on B cells, suggesting that B cells contribute to the prominent inflammatory response in this disease. Further mechanistic studies are required to determine the B cell subsets overexpressing TLRs in Behçet’s disease.

## 4. Potential Therapeutic Avenues for Autoinflammatory Disorders

Neuro-Behçet’s disease (NBD) is a multisystem auto-inflammatory disorder characterized by an inflammatory cascade and infiltration of the cerebrospinal fluid (CSF) by neutrophils [48]. In studies of NBD, increased levels of CXCL9/CXCL10 chemokines were found to be associated with B cell migration [49], and the longevity factor BAFF (B cell activating factor) was reported to be upregulated in NBD patients [50]. More recently, the contribution of IL-10-producing cells present in the peripheral blood and CSF of treatment-naive patients suffering from NBD was investigated and compared to patients affected with remitting-relapsing multiple sclerosis. Strikingly, B cells present in the CNS were the major source of intrathecal IL-10 in NBD [51]. It is possible that, in addition to IL-10, B cells present in the CSF produce other cytokines that play a proinflammatory role, i.e., TNF-α, IL-6, and GM-CSF. Remarkably, B cell depletion therapy was reported to be effective in severe ocular manifestations of Behçet’s disease [52], in two cases of neuro-Behçet disease presenting pseudo-tumoral lesions [53], and in one case with relapsing NBD [54]. It is tempting to speculate that B cells represent a promising therapeutic target in patients with NBD.

Neuromyelitis optica spectrum disorder (NMOSD) is an inflammatory demyelinating disorder of the central nervous system (CNS). The disorder affects predominantly CNS astrocytes, leading to astrocytopathy. It can progress to blindness, paralysis, serious disability, and even mortality. Patients often have IgG autoantibodies directed to the water channel protein aquaporin-4, and detection of this antibody subset in the serum of patients facilitates the diagnosis of NMOSD [55]. B cells play an important role in the pathogenesis, with production of memory and autoreactive B cells. B cell depletion therapy is effective in NMOSD patients despite the fact that it does not result in a significant reduction in serum autoantibody levels, indicating that additional mechanisms are implicated, including antibody-independent functions of B cells [56,57]. In addition, there are alterations of regulatory B cells in NMOSD patients. Treatment strategies for acute attacks of NMOSD include intravenous immunoglobulins, methylprednisolone, plasmapheresis and immuno-adsorption [58]. Relapses of NMOSDs can be prevented by conventional immunosuppressants, B cell-depleting agents, IL-6 signaling blocking drugs, and agents interfering with complement components. It is of note that some common inflammatory systemic diseases can exhibit ocular inflammation, including spondyloarthropathies, psoriatic arthritis, ulcerative colitis, Crohn’s disease and Behçet’s syndrome. It is therefore likely that both innate autoinflammation and autoimmune phenomena are required for full clinical expression of such non-infectious uveitic conditions.

Humans and mice with mutations in the *AIRE* gene suffer from a syndrome with multiorgan autoinflammatory infiltrates. In a mouse model of *AIRE*-deficient patients, B cells were reportedly required for fulminant multiorgan inflammation [59]. Presumably, B lymphocytes could promote pathology through different mechanisms, including influencing T cell function by regulation of their activation status. These observations raise the possibility that targeting B cells could be beneficial in *AIRE*-deficient patients who develop autoimmune polyendocrinopathy-candidiasis-ectodermal dystrophy (APECED).

## 5. Multiple B Cell-Mediated Modulatory Effects in Autoinflammatory Disorders

**Figure 2:**
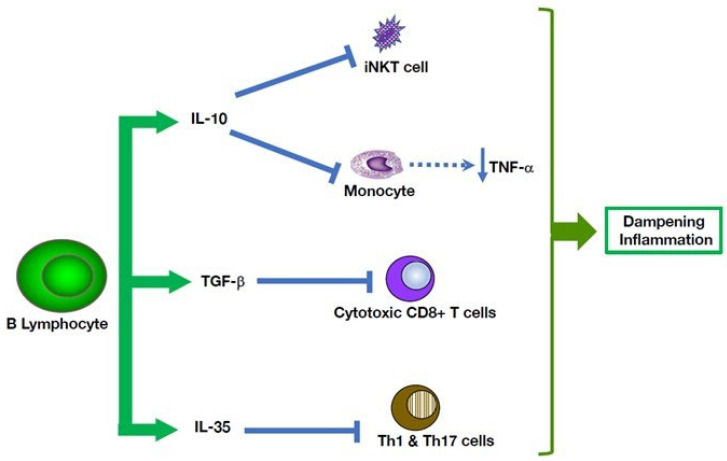
B cell contact-dependent mechanisms that modulate innate immunity. B cell modulating activities are not limited to cytokine production. Co-stimulatory interactions with dendritic cells or T cells can lead to immune suppression. In the innate branch of the immune system, B cells can modulate expression of invariant natural killer T (iNKT) cells through presentation of lipid antigens via the CD1d receptor, leading to expansion of inflammatory INF-gamma production.

Beyond immunoglobulin secretion, B lymphocytes play chief roles in the adaptive immune system, such as antigen presentation to T cells and development of secondary lymphoid organs [56,60]. In addition to their well-established effector functions (Figure 1), B lymphocytes exert functions that prevent uncontrolled inflammation [61]. Remarkably, a subset of B cells acts in negative regulation of the immune system by mitigating inflammatory responses (Figure 2). The B cells exert this immunomodulatory role mainly through cytokine production of anti-inflammatory mediators, such as IL-10, TGF-β and IL-35, and their expansion can be advantageous in chronic inflammatory conditions [61]. Induction of this suppressive B cell phenotype requires a combination of signals that include activation through the B cell receptor, CD40, co-stimulatory molecules, i.e., CD80 and CD86, inflammatory cytokines, and toll-like receptors.

**Figure 3:**
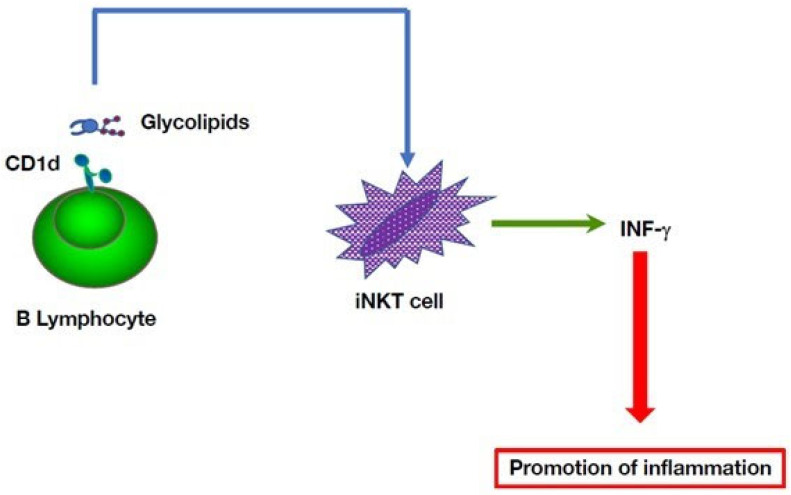
B cells produce cytokines that can mitigate various pro-inflammatory pathways. Anti-inflammatory activities of B cells that act on cells of the innate immune system have been well documented. Thus, human B cells can exert multiple immune suppressive activities on cells of both the adaptive and innate immune systems. They can inhibit CD8+ T cell-mediated cytotoxicity and suppress Th1 and Th17 inflammatory responses. In parallel, they also promote the differentiation of CD4+ T cells into IL-10+ T regulatory-1 (Tr1) cells and FoxP3+ Tregs cells. In addition to impacting adaptive immune responses, B cells reduce TNF-α production by monocytes, and reduce functions of iNKT cells.

In parallel, B cell regulatory mechanisms implicating direct cell-cell communications have been reported. An important suppressive pathway targets invariant Natural Killer T cells (iNKT). This T cell subset is characterized by early and rapid cytokine responses upon activation. It recognizes specifically glycolipids in the context of the non-polymorphic, MHC class I-like antigen presenting molecule CD1d [62]. According to their cytokine production potential and transcription factor expression, iNKTs are categorized in several functional subsets, i.e., iNKT1, iNKT2, iNKT17, iNKTFH, and iNKT10 [62]. Importantly, B lymphocyte populations express CD1d to some degree. In humans, MZ B cells express high levels of CD1d, and murine B-1 cells also are highly positive for CD1d, which enables them to load foreign lipids, and even self-lipids during sterile inflammation, on this receptor (Figure 3). Through this mechanism, B cells can trigger IFN-g production by iNKT cells, thereby modifying the balance of cytokine secretion by iNKT cells and lowering Th1 and Th17 type responses. Experiments using mice specifically deficient in CD1d on B cells support this view [63].

**Figure 4:**
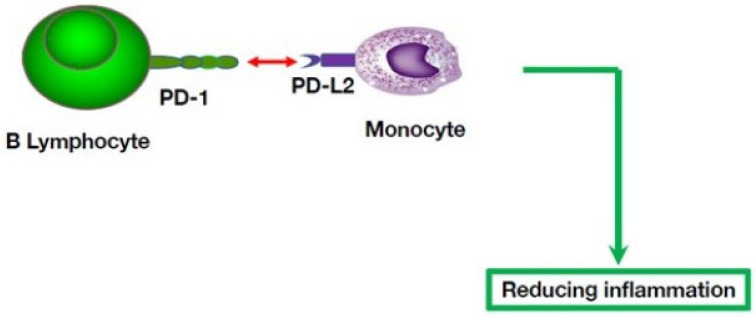
B cell contact-dependent mechanisms modulating innate immunity by inhibitory receptors expressed by B cells. B cells can contact and suppress other effector cells through inhibitory receptors, including PD-1. The immunosuppressive PD-1 receptor is expressed by B lymphocytes, including germinal center B cells and plasma cells [65,66], and its PD-L1 and PD-L2 receptors are expressed by non-lymphoid cells, including monocytes and macrophages. Thus, this pathway represents another regulatory mechanism that enables B cells to impact a key player of innate immunity, i.e., the monocyte.

Another immuno-suppressive potential of B cells is independent of IL-10, but mediated by the programmed cell death (PD-1) pathway [64]. Specifically, populations of B lymphocytes that include marginal zone (MZ)-progenitor B cells and MZ-like B cells express the programmed cell death-ligand 1 (PD-L1) molecule, and this PD-1/PD-L1 pathway can modulate follicular T-helper (Tfh) cell responses. On the other hand, monocytes express another PD-1 receptor, i.e., PD-L2, and their interactions with B cells expressing PD-1 can lead to reduction of the innate functions of monocytes Figure 4).

## 6. Future Prospects

**Figure 5:**
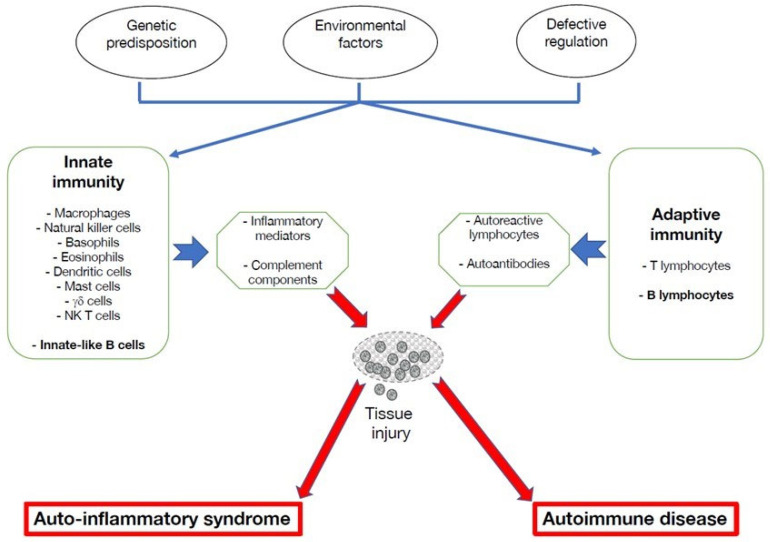
Genetic predisposition, environmental factors, and defective immune regulation underlie initiation of both autoimmune diseases and auto-inflammatory syndromes. The BLK and CD40 loci are known to be associated with AIDs, such as lupus, arthritis and Crohn’s disease, as well as AIFS, such as Kawazaki syndrome. Environmental triggers have been documented in several AID and AIFS. Defects in development or functions of cells of both innate and adaptive immunity may lead to abnormal functionalities in these cells and altered or hyperactive immune responses [56,70]. These various factors, in isolation or in combination, can result in tissue injury. By virtue of their roles in both the innate and adaptive immune systems, B cells are at the crossroads of autoimmune diseases and auto-inflammatory syndromes.

The view that autoimmune diseases are triggered by aberrant adaptive immune responses, and that AIFS are caused solely by alterations in the innate branch the immune system that lead to tissue inflammation and injury, with little or no involvement of T and B cells [3,67], needs to be revised. As discussed above, the underlying triggers of both auto-inflammation and autoimmunity remain to be identified. Given the multiple cross-talks between both branches of innate and adaptive immunity in mounting coordinated signals, there is a significant overlap between the trajectories that lead to autoimmune diseases or AIFS, as depicted in Figure 5.

Whereas AIFS are characterized by hyper-reactivity of the innate arm of the immune system, the contribution of lymphocytes has been documented in different diseases. By virtue of their potential to play a role in adaptive immunity and to exert innate-like functions [68,69], B cells can be involved in both promoting inflammation or mitigating auto-inflammation in AIFS. Since autoimmunity and autoinflammation engage distinct molecular pathways, a more detailed understanding of their respective roles in the development of inflammation could lead to designing novel therapeutic targets.

## Data Availability

Not required.
